# Screen‐Detected Coeliac Disease in a Nationally Representative Cohort: Health Indicators, Long‐Term Outcomes and Mortality

**DOI:** 10.1111/apt.70917

**Published:** 2026-08-10

**Authors:** Iida Ahonen, Kalle Kurppa, Heini Huhtala, Katri Kaukinen, Laura Kivelä, Juha Taavela

**Affiliations:** ^1^ Celiac Disease Research Centre Tampere University Tampere Finland; ^2^ Tampere Centre for Child, Adolescent and Maternal Health Research Tampere University Tampere Finland; ^3^ Department of Pediatrics Tampere University Hospital, Well‐Being Services County of Pirkanmaa Tampere Finland; ^4^ The University Consortium of Seinäjoki Seinäjoki Finland; ^5^ Faculty of Social Sciences University of Tampere Tampere Finland; ^6^ Department of Internal Medicine Tampere University Hospital, Well‐Being Services County of Pirkanmaa Tampere Finland; ^7^ Children's Hospital and Pediatric Research Centre University of Helsinki and Helsinki University Hospital Helsinki Finland; ^8^ Department of Gastroenterology and Alimentary Tract Surgery Tampere University Hospital, Well‐Being Services County of Pirkanmaa Tampere Finland

**Keywords:** coeliac disease, gluten‐free, population, quality of life, serology, testing

## Abstract

**Background:**

Population‐based screening for coeliac disease could reduce underdiagnosis, but benefits and harms remain debated.

**Aim:**

We studied health outcomes in screen‐detected coeliac disease patients at diagnosis and following a long‐term gluten‐free diet.

**Methods:**

Data were collected from Health 2000 and 2011 studies representing the Finnish population. Coeliac disease was screened in 6339 individuals by transglutaminase and endomysial antibodies, with dual seropositivity as the diagnostic criterion, histologically validated in this cohort. Survey‐weighted analyses enabled population‐level results. The follow‐up cohort comprised those participating in both time points. Mortality was assessed between 2000 and 2017.

**Results:**

At screening, 92 (1.5%) undiagnosed coeliac disease patients and 6216 non‐coeliac controls were identified. Patients were less often smokers (11% vs. 22%, *p* = 0.014), more often had a history of pre‐eclampsia (13% vs. 5%, *p* = 0.037) and anaemia (14% vs. 6%, *p* = 0.012), and lower ferritin (29 vs. 68 ng/mL, *p* < 0.001), with an altered lipid profile including lower total cholesterol, LDL‐C, and ApoB but higher atherogenic ratios. At 11‐year follow‐up, mental health, quality of life, and cardiometabolic markers were comparable between treated coeliac disease patients (*n* = 50) and non‐coeliac controls (*n* = 3226). Mortality did not differ after adjustment for age and sex (HR 0.77, 95% CI 0.45–1.33).

**Conclusions:**

Nationally screened adults with undiagnosed coeliac disease showed iron deficiency and anaemia. Long‐term dietary treatment did not adversely affect quality of life or cardiometabolic health and may prevent complications. Furthermore, mortality was comparable to the general population. The results suggest a favourable benefit‐harm balance for population‐based screening.

## Graphical abstract

In a nationally representative Finnish cohort, screen‐detected coeliac disease was associated with iron deficiency, anaemia, and an atherogenic lipid profile at diagnosis, but long‐term gluten‐free treatment did not adversely affect quality of life, metabolic health, or mortality; supporting a favourable benefit‐harm balance for population‐based coeliac disease screening.

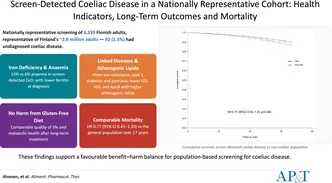

## 1 Introduction

Coeliac disease (CeD) is a common autoimmune condition induced by gluten ingestion in individuals with genetic susceptibility [1, 2]. The presentation of the disease has changed over the decades towards milder forms, and today, many patients have atypical symptoms or are even identified as apparently asymptomatic [3]. The diverse clinical presentation makes symptom‐based case‐finding strategies challenging and results in missed diagnoses and diagnostic delays despite increasing awareness and improved diagnostic tools [2, 4, 5, 6, 7]. While screening for CeD in at‐risk groups is currently recommended in many guidelines [8, 9, 10, 11], population screening has not been considered justified, though it remains under debate [12].

The World Health Organisation guidelines for population screening outline specific requirements, many of which CeD meets; however, evidence that screening benefits outweigh harms, particularly regarding long‐term treatment outcomes at the population level, remains limited [13, 14]. Especially asymptomatic patients may have less motivation to adhere to a restrictive gluten‐free diet, with attendant risk of declining quality of life [15]. Moreover, although symptomatic clinically identified CeD patients face a confirmed risk of various complications when untreated [16, 17, 18], the risk profile is less well‐defined among screen‐detected, often asymptomatic patients. Prevention of complications is an important consideration in screening, as it may provide not only clinical benefits for patients but also a significant cost‐effectiveness advantage.

The aim of the study was to investigate general health characteristics at diagnosis and after a long‐term gluten‐free diet in CeD patients identified through screening within a population‐representative cohort. Additionally, mortality was compared between CeD patients and the general population over a 17‐year follow‐up period.

## 2 Methods

### 2.1 Study Design

The study was conducted in collaboration with the Finnish Institute for Health and Welfare. It was based on two nationally representative health surveys carried out by the Institute in 2000 and 2011 whose main aims were to provide up‐to‐date data on major public health problems in Finland [19, 20]. Adults aged ≥ 30 years and living in Finland were invited to participate in the Health 2000 survey, and all living members of the original Health 2000 survey were re‐invited to participate in the Health 2011 survey. The surveys comprised a two‐stage stratified cluster sampling and weighting scheme that estimates health statistics representative of the same‐aged Finnish population [19, 21].

To facilitate two‐stage stratified sampling, the sample frame was stratified regionally according to five university hospital regions: namely those of Helsinki, Turku, Tampere, Kuopio, and Oulu. In the first stage of sampling, 80 health centre districts were sampled as clusters. The sample size for each health centre district was proportional to its proportion of population. In the second stage, systematic random sampling was used to draw the sample from each health centre district using the nationwide population register. The unweighted participation rate at any stage of the survey was 93% in the Health 2000 and 73% in the Health 2011 [19, 20]. In total, the surveys comprised 8028 and 8177 individuals, forming population‐representative samples for 3,230,382 and 3,254,681 Finnish adults, respectively. The total population of Finland considering all ages was 5,181,115 in 2000 and 5,401,267 in 2011 [22].

Survey participants went through health examinations, interviews, and blood sampling, and completed health questionnaires. Interviews were performed either at a separate home visit, at local healthcare facilities or by phone. Health examinations were conducted by a physician during the home visit or at the local healthcare facility afterwards. Blood samples for research purposes were collected during the visits and stored frozen at −70°C for later use. Data from patient data registries were obtained to supplement the medical information. Furthermore, mortality data for the Health 2000 cohort were acquired from national statistics registry after 17‐year follow‐up.

### 2.2 Clinical Data

Demographic factors, healthcare utilisation, and lifestyle factors such as smoking status and use of alcohol were collected with questionnaires and interviews. Presence of chronic medical conditions, reproductive health factors and current gluten‐free diet use were obtained via self‐reported questionnaires or patient registry data. Blood samples were collected for a broad spectrum of laboratory measurements, including haemoglobin, ferritin, vitamin D, liver enzymes and lipid parameters, and handled using a standardised protocol. Detailed descriptions of the study protocols have been published previously [19, 21]. Anaemia was defined as haemoglobin < 115 g/L in women and < 132 g/L in men. Additionally, physical measurements including body mass index (kg/m^2^), waist circumference (cm) and blood pressure (mmHg) were recorded at study visits.

The assessment of depression, anxiety and general psychological distress was carried out using the validated General Health Questionnaire (GHQ‐12) at both time points and Beck Depression Inventory (BDI) in 2000. For measuring quality of life, EUROHIS‐QOL was used in 2011. GHQ‐12 is a 12‐item questionnaire and the scores are based on a 4‐point Likert scale (0–1–2–3), with responses to all items summed up to produce a total score ranging from 0 to 36, with higher scores indicating more psychological distress [23]. BDI is a 21‐item, multiple‐choice inventory employing Guttman scaling designed to assess the level of depression in adults. Each item is scored from 0 to 3 points, with the total score ranging from 0 to 63, with higher scores indicating more depressive symptoms [24]. EUROHIS‐QOL is an 8‐item index [25] taken from the WHOQOL‐BREF [26] with a 5‐point scale; the total score is the sum of all items. Higher scores indicate better quality of life.

Serum IgA class transglutaminase antibodies (TGA) and endomysial antibodies (EmA) were analysed at Tampere University Celiac Disease Research Centre by commercial Eu‐tTG (Eurospital S.p.A., Trieste, Italy; Health 2000) or Celikey (Phadia, Freiburg, Germany; Health 2011) assay. Both assays use human recombinant transglutaminase as an antigen, and the results are given in arbitrary units. Values ≥ 7.0 U/mL for Eu‐tTG and ≥ 5.0 U/mL for Celikey were considered positive and further tested for EmA. EmA were determined using an indirect immunofluorescence method as described elsewhere [27]. A serum dilution of 1:≥ 5 was considered positive and a positive sample was further diluted up to 1:4000.

### 2.3 Study Participants

Only individuals who had provided sera for the CeD autoantibody analyses were included in the current study (Figure 1). Those with previous CeD diagnosis and/or following a gluten‐free diet were excluded. A CeD patient was defined as an individual with positivity to both TGA and EmA regardless of antibody levels. A non‐CeD subject was defined as an individual with negative CeD serology. Those with positive TGA but negative EMA were excluded from further analyses due to this serological discordance, given the highly specific nature of EMA and the correspondingly low probability of CeD in this group.

**FIGURE 1:**
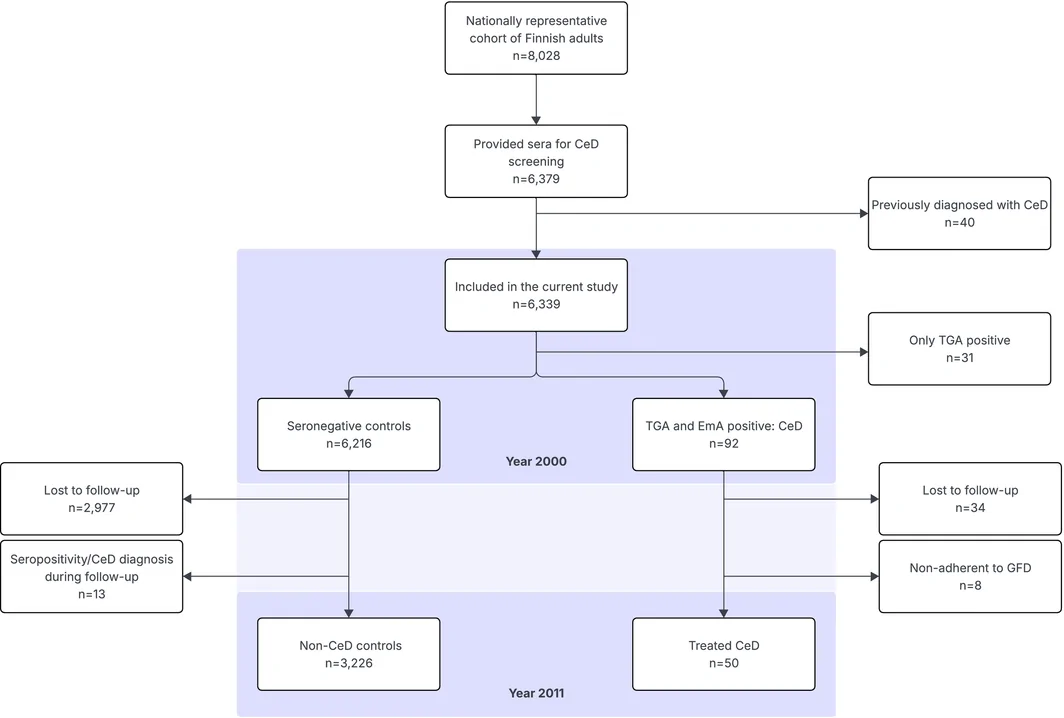
Flowchart of the study. The sera were assessed for tissue transglutaminase (TGA) and endomysial antibodies (EmA), and individuals positive for both antibodies were considered as having screen‐detected coeliac disease (CeD). GFD, gluten‐free diet.

Initial CeD screening was performed in 2000 and baseline characteristics were compared between screen‐detected CeD patients and the non‐CeD population. The follow‐up study cohort consisted of subjects who also attended the investigations in 2011. CeD patients not reporting to follow a gluten‐free diet in 2011 and non‐CeD subjects diagnosed with CeD during follow‐up or having positive TGA and/or EmA in 2011 testing were excluded from the follow‐up evaluations (Figure 1).

Subjects with positive CeD antibody levels in the sera obtained via the study were contacted and referred to healthcare to receive appropriate diagnostic evaluation and follow‐up. A previous study using the same material showed that contacts via the study led to small‐bowel biopsies in approximately half of the seropositive subjects of whom 75% received a biopsy‐proven diagnosis of CeD [28]. Due to Finnish data protection regulations, biopsy data could not be directly accessed in the current study. However, double seropositivity for TGA and EmA is regarded as highly specific for CeD and has been validated against biopsy‐proven diagnosis in large screening studies [28, 29].

### 2.4 Statistics

Descriptive data are given as percentages or as median and interquartile ranges. Statistical comparisons between the study groups were carried out with either Chi‐square test or Mann–Whitney *U* test. Analysis of covariance and logistic regression were used to adjust for age and sex, and smoking status as appropriate, between the groups. Mortality was analysed using Cox regression with adjustments for age and sex. To obtain nationally representative data for the 2000 cohort, a complex samples analysis function with provided weights was applied. The inverse probability weights were used to reduce bias due to non‐participation and to provide nationally representative results. Post‐stratification weights were calibrated according to age, sex, area and language in 2000. *p* < 0.05 were considered significant. All analyses were executed with the Statistical Package for the Social Sciences (SPSS), version 29.0 (IBM, Armonk, NY, USA).

## 3 Results

In total, 92 (1.5%) new CeD patients were identified from population screening of 6339 individuals, representing 43,475 CeD adults out of 2,915,925 Finnish adults (Figure 1). Median TGA levels among patients were 18.6 U/mL (IQR 14.5–21.9, range 18.1 U/mL). Compared to non‐CeD individuals, newly identified CeD patients were less often regular smokers, had higher rates of type 1 diabetes, psoriasis, and history of pre‐eclampsia (Table 1, Figure 2). The groups did not differ significantly in age, sex, education level, healthcare utilisation, medication use, other assessed lifestyle factors, chronic medical conditions, reproductive health factors, or physical measurements (Table 1, Figure 2).

**TABLE 1:** Demographics, health care utilisation, lifestyle factors, physical measurements and psychological health in screen‐detected coeliac disease (CeD) patients compared to non‐CeD population at baseline in 2000.

	CeD *N* = 92	Non‐CeD *N* = 6216	
	Population representative sample		
	*N* = 43,475	*N* = 2,872,450	*p* ^a^
Demographics			
Age, year, median (Q1, Q3)	49 (42, 58)	51 (41, 62)	0.252^b^
Female, %	62	53	0.149^b^
Education level, %			
Basic^c^	39	40	0.258^b^
Secondary^d^	39	32	
Higher^e^	22	28	

Health care utilisation^f^	%	%	
At least one doctor's visit	72	74	0.409
Use of mental health services	3	6	0.276
Lifestyle			
Smoking regularly	11	22	**0.006**
Smoking at least for 1 year	42	55	**0.043**
Alcohol consumption, g/week, median (Q1, Q3)	14 (0, 55)	23 (0, 89)	0.136
Use of prescription medicine^f^	74	79	0.193
Use of non‐prescription medicine^f^	83	80	0.911

Physical measurements	Median (Q1, Q3)	Median (Q1, Q3)	
Body mass index, kg/m^b^	26 (24, 29)	26 (24, 30)	0.495
Waist circumference, cm	90 (82, 98)	93 (83, 102)	0.313
Systolic blood pressure, mmHg	131 (118, 144)	132 (120, 148)	0.544
Diastolic blood pressure, mmHg	81 (72, 88)	82 (74, 90)	0.193
Psychological health			
GHQ‐12, total score	0 (0, 2)	0 (0, 2)	0.102
BDI, total score	4 (1, 8)	5 (2, 10)	0.110

*Note:* Bolded *p* values denote statistical significance.

Abbreviations: BDI, beck depression inventory, higher score indicates more depressive symptoms; GHQ‐12, general health questionnaire, higher score indicates more psychological distress.

^a^ Weighted and adjusted for age and sex.

^b^ Only weighted.

^c^ Not completed vocational school, matriculation examination or degrees from higher vocational institutions, polytechnics and universities.

^d^ Completed vocational school and/or passed matriculation examination.

^e^ Degrees from higher vocational institutions, polytechnics and universities.

^f^ During previous 12 months.

**FIGURE 2:**
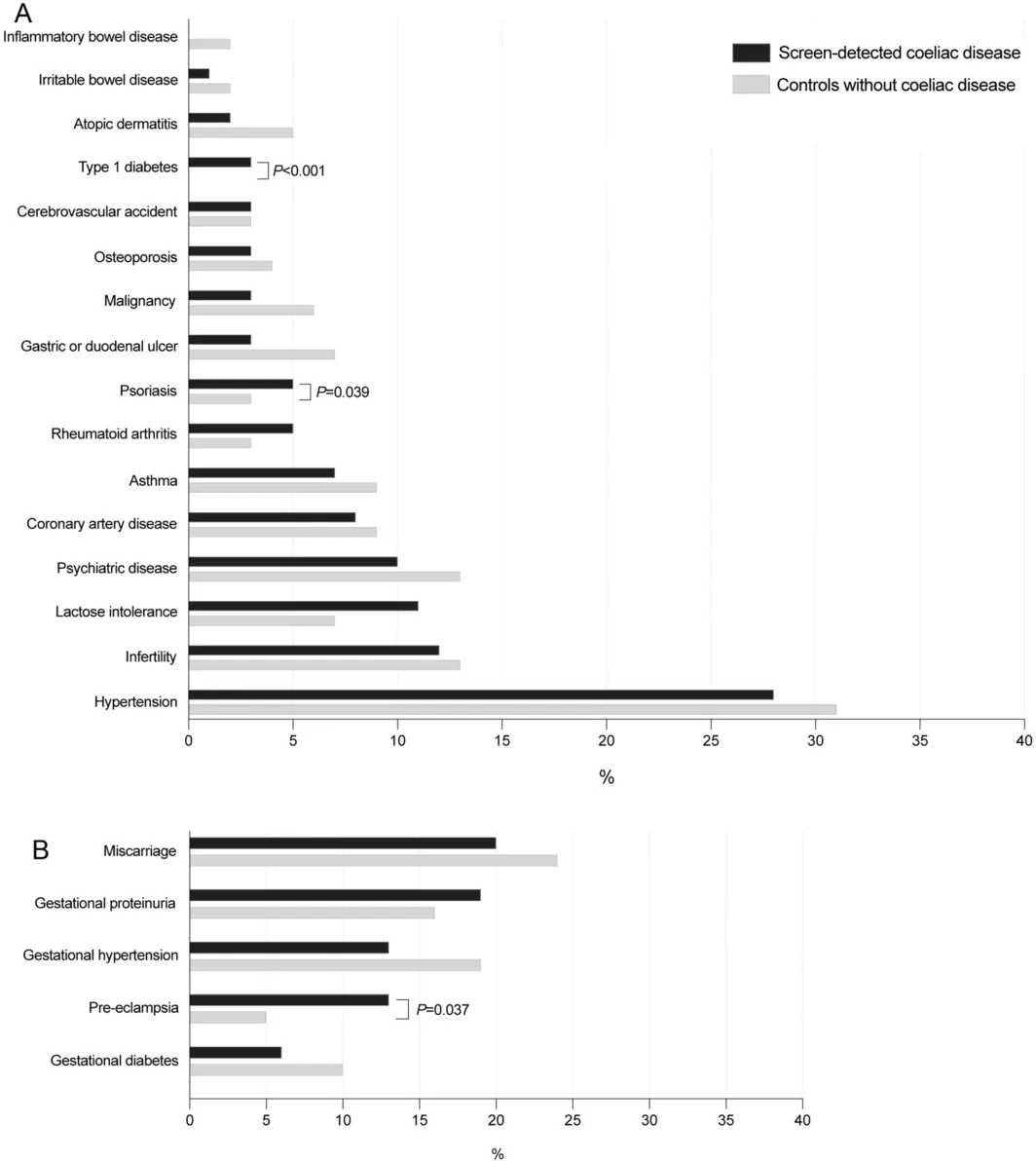
Panel A shows chronic medical conditions in screen‐detected coeliac disease patients (*n* = 92, representing 43,475 Finnish adults) and controls without coeliac disease (*n* = 6216, representing 2,872,450 Finnish adults) at initial screening in 2000. Only *p* values that remained significant after weighting and adjustment for age and sex are shown. Panel B shows reproductive health among women with a history of pregnancy, assessed at initial screening in 2000, comparing screen‐detected coeliac disease patients (*n* = 92) with controls without coeliac disease (*n* = 2933). Only significant *p* values after adjustment for type 1 diabetes and smoking status are shown.

Anaemia was more prevalent among the newly identified CeD patients compared to the non‐CeD population (14% vs. 6%, *p* < 0.001 in weighted and age‐ and sex‐adjusted analysis). Haemoglobin, ferritin, gamma‐glutamyl transferase, creatinine, and most lipid parameters were lower among CeD patients in weighted and age‐ and sex‐adjusted analyses; triglycerides were comparable between the groups (Table 2). No significant differences between the groups were seen in other laboratory parameters (Table 2). Adjusting for smoking status did not alter the results (data not shown). Self‐reported current health status was similar between CeD patients and the non‐CeD population (‘good’ or ‘fairly good’: 66% vs. 62%, *p* = 0.456 in weighted and age‐ and sex‐adjusted analysis). Furthermore, there was no difference in GHQ‐12 or BDI total scores between the groups (Table 1). Of the parameters evaluated at baseline, results from crude analyses and age‐ and sex‐adjusted analyses without weighting are displayed in Table S1. These results mainly aligned with those from weighted analysis.

**TABLE 2:** Laboratory parameters in screen‐detected coeliac disease (CeD) patients compared to non‐CeD population at baseline in 2000.

	CeD *N* = 92	Non‐CeD *N* = 6216	*p* ^a^
	Population representative sample		
	*N* = 43,475	*N* = 2,872,450	
	Median (Q1, Q3)	Median (Q1, Q3)	
Haemoglobin, g/L	140 (129, 150)	143 (134, 153)	**0.012**
Ferritin, ng/mL	30 (10, 78)	70 (33, 131)	**0.004**
GGT, U/L	19 (15, 30)	24 (17, 39)	**0.044**
S‐D‐25, nmol/L	38 (31, 52)	43 (33, 55)	0.537
Creatinine, μmol/L	68 (60, 75)	71 (63, 81)	**0.042**
TSH, mU/L	1.4 (0.9, 2.0)	1.4 (1.0, 2.0)	0.537
Lipid parameters			
TC, mmol/L	5.4 (4.7, 5.9)	5.9 (5.2, 6.6)	**< 0.001**
HDL‐C, mmol/L	1.1 (0.9, 1.3)	1.3 (1.1, 1.6)	**< 0.001**
sdLDL‐C, mmol/L	3.3 (2.8, 4.2)	3.7 (3.0, 4.4)	**0.016**
TC/HDL‐C ratio	5.1 (3.8, 6.3)	4.5 (3.6, 5.7)	0.093
TG, mmol/L	1.2 (0.9, 1.7)	1.3 (1.0, 1.9)	0.626
ApoA1, g/L	1.4 (1.2, 1.5)	1.6 (1.4, 1.8)	**< 0.001**
ApoB, g/L	1.2 (0.9, 1.3)	1.2 (1.0, 1.4)	**0.011**
ApoB/ApoA1 ratio	0.9 (0.7, 1.0)	0.8 (0.6, 1.0)	**0.020**

*Note:* Bolded *p* values denote statistical significance.

Abbreviations: A1; ApoB, serum apolipoprotein B; ApoA1, serum apolipoprotein; GGT, gamma‐glutamyl transferase; HDL‐C, serum high‐density lipoprotein cholesterol; S‐D‐25, serum 25‐hydroxyvitamin D; sdLDL‐C, serum small dense low‐density lipoprotein cholesterol; TC, serum total cholesterol; TG, serum triglycerides; TSH, thyroid stimulating hormone.

^a^ Weighted and adjusted for age and sex.

Fifty screen‐detected CeD patients on a gluten‐free diet and 3226 non‐CeD individuals participated in the follow‐up study (Figure 1). Among the follow‐up cohort, a lower proportion of subjects had a higher education level when compared to the baseline cohort, but no other relevant differences were seen between the cohorts. Comparison of background characteristics between baseline and follow‐up cohorts at initial screening in 2000 is shown in Table S2. After 11 years, healthcare utilisation, enquired lifestyle factors including smoking status, and physical measurements were comparable between CeD patients and the non‐CeD population (Table 3). CeD patients had lower levels of total cholesterol, triglycerides and apolipoprotein B compared with the non‐CeD population, while other laboratory parameters did not differ between the groups (Table 3). Notably, haemoglobin and ferritin levels could not be assessed at follow‐up, as these parameters were not collected in the Health 2011 survey. Self‐reported current health status (‘good’ or ‘fairly good’: 82% vs. 76%, *p* = 0.405) was similar between CeD patients and non‐CeD controls, and there was no difference in GHQ‐12 or EUROHIS‐QOL total scores between the groups (Table 3). Furthermore, in a subgroup analysis of screen‐detected patients following (*n* = 50) and not following (*n* = 8) a gluten‐free diet, self‐reported health status and the results of the GHQ‐12 and EUROHIS‐QOL were comparable, though the small sample size limits the conclusions that can be drawn here (Table S3). Levels of TGA had normalised in all CeD patients following a gluten‐free diet. Of those individuals who were TGA‐positive but EMA‐negative at initial screening (*n* = 12), 25% had received a physician‐reported CeD diagnosis during the follow‐up period.

**TABLE 3:** Health care utilisation, lifestyle factors, physical measurements and laboratory parameters after 11‐year follow‐up in screen‐detected coeliac disease (CeD) patients (*n* = 50) on a gluten‐free diet compared to non‐CeD population (*n* = 3226).

	CeD *N* = 50	Non‐CeD *N* = 3226	*p* ^a^	*p* ^b^
Health care utilisation (%)^c^				
At least one doctor's visit	58	71	0.061	0.063
Lifestyle				
Smoking regularly	12	13	1.000	0.709
Use of prescription medicine^c^	90	83	0.253	0.179
Use of non‐prescription medicine^c^	68	73	0.523	0.373
Use of vitamin or mineral supplement^c^	72	65	0.370	0.315

Physical measurements	Median (Q1, Q3)	Median (Q1, Q3)		
Body mass index, kg/m^b^	26 (25, 30)	27 (24, 30)	0.895	0.428
Waist circumference, cm	92 (85, 102)	93 (84, 102)	0.937	0.527
Systolic blood pressure, mmHg	131 (120, 149)	134 (122, 146)	0.920	0.843
Diastolic blood pressure, mmHg	80 (76, 88)	80 (74, 88)	0.608	0.966
Laboratory parameters				
ALAT, U/L	20 (16, 29)	21 (16, 29)	0.634	0.278
GGT, U/L	20 (15, 40)	24 (16, 38)	0.236	0.375
Creatinine, μmol/l	72 (63, 81)	72 (63, 82)	0.911	0.952
S‐D‐25, nmol/L	78 (70, 93)	73 (61, 87)	0.053	0.070
TC, mmol/L	5.2 (4.4, 5.9)	5.4 (4.8, 6.1)	**0.036**	**0.006**
HDL‐C, mmol/L	1.5 (1.2, 1.7)	1.5 (1.3, 1.8)	0.526	0.298
LDL‐C, mmol/L	3.3 (2.4, 3.8)	3.2 (2.7, 3.9)	0.220	0.072
TC/HDL‐C ratio	3.5 (2.9, 4.0)	3.5 (3.0, 4.3)	0.386	0.120
TG, mmol/L	1.0 (0.7, 1.3)	1.1 (0.9, 1.6)	**0.021**	**0.033**
ApoA1, g/L	1.5 (1.4, 1.7)	1.5 (1.4, 1.7)	0.400	0.416
ApoB, g/L	0.9 (0.7, 1.0)	0.9 (0.8, 1.0)	**0.049**	**0.008**
ApoB/ApoA1 ratio	0.6 (0.4, 0.7)	0.6 (0.5, 0.7)	0.205	0.053
Psychological health				
GHQ‐12, total score	0 (0, 1)	0 (0, 2)	0.247	0.786
EUROHIS‐QOL, total score	33 (30, 35)	33 (29, 35)	0.591	0.862

*Note:* Bolded *p* values denote statistical significance.

Abbreviations: ALAT, alanine aminotransferase; ApoA1, serum apolipoprotein A1; ApoB, serum apolipoprotein B; EUROHIS‐QOL, higher score indicates better quality of life; GGT, gamma‐glutamyl transferase; GHQ‐12, general health questionnaire, higher score indicates more psychological distress; HDL‐C, serum high‐density lipoprotein cholesterol; LDL‐C, serum low‐density lipoprotein cholesterol; S‐D‐25, serum 25‐hydroxyvitamin D; TC, serum total cholesterol; TG, serum triglycerides.

^a^ Crude analysis.

^b^ Adjusted for age and sex.

^c^ During previous 12 months.

The age‐ and sex‐adjusted mortality rate, evaluated 17 years after the baseline investigations, was comparable between CeD patients and the non‐CeD population (14% vs. 25%; HR 0.77, 95% CI 0.45–1.33, *p* = 0.350) (Figure 3). Of those CeD patients who participated in follow‐up investigations in 2011 and did not follow a gluten‐free diet (*n* = 8), none had died between 2011 and 2017.

**FIGURE 3:**
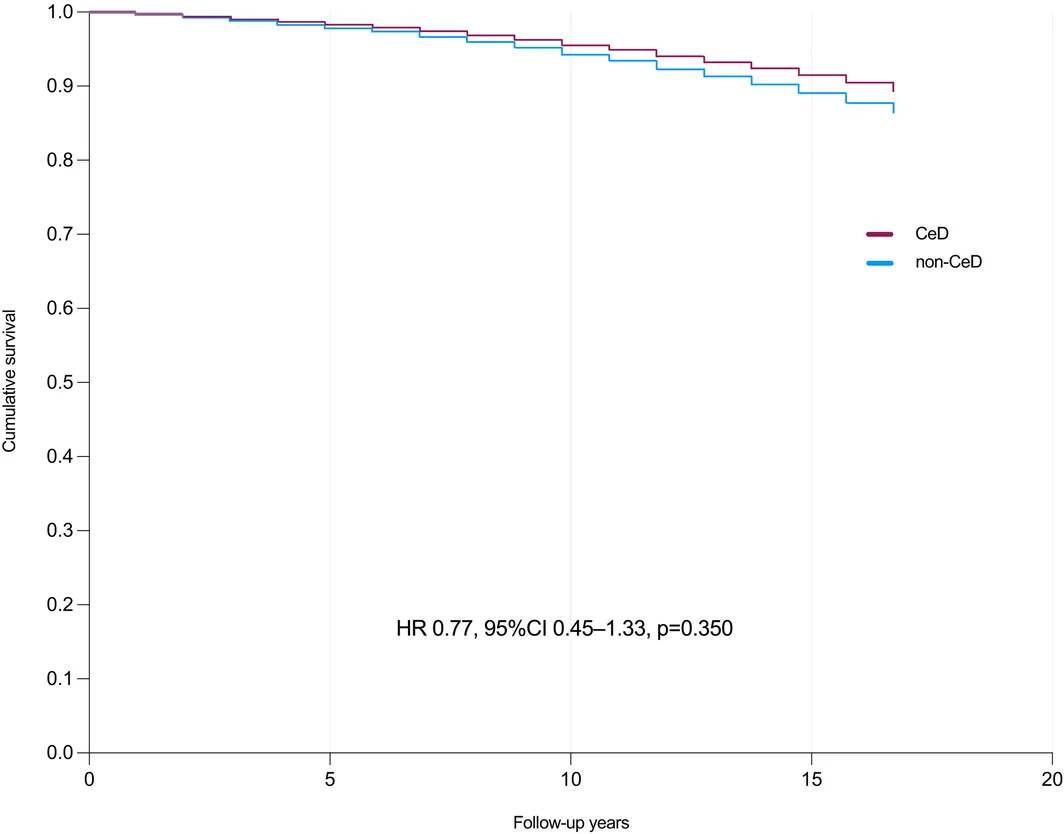
Cumulative survival in screen‐detected coeliac disease (CeD) patients (*n* = 92) and non‐CeD population (*n* = 6216), analysed with Cox regression and adjusted for age and sex.

## 4 Discussion

In this large screen‐detected cohort, undiagnosed adult CeD patients exhibited signs of active disease, including iron deficiency and an overrepresentation of anaemia, and a gluten‐free diet was not associated with detrimental effects on long‐term quality of life or markers of cardiometabolic health. Furthermore, a considerable proportion of patients appears to remain undetected when relying on currently recommended case‐finding and risk‐group approaches. These findings offer support for population‐based screening, though the CeD‐specific burden of life‐long dietary treatment, along with the costs of population‐based screening, warrants further detailed analysis.

The excess of iron deficiency and anaemia among CeD patients compared with the non‐CeD population is consistent with expectations, as these are hallmark features of untreated CeD, reflecting impaired intestinal absorption [30]. The observation aligns with findings from previous population‐based screening studies and improvement in ferritin and haemoglobin levels during dietary treatment has been seen among this patient group [30, 31, 32, 33]. Importantly, these findings support the idea that the disease among screen‐detected patients is not biologically different from that found by other diagnostic approaches. In addition, there is a concern that anaemia may be associated with more severe disease [32, 33], further underlining the importance of identifying these patients through screening. Patients with the most pronounced symptoms, however, are typically identified clinically before screening reaches them, so the spectrum of clinical findings among screen‐detected patients may be generally milder, which is also consistent with the absence of individuals having TGA levels > 10 times the upper limit of normal in our cohorts.

The most notable additional laboratory differences were observed in lipid profiles. The concurrently lower levels of LDL, HDL, ApoB and ApoA1 in CeD patients, together with the comparable BMI, may partly reflect impaired intestinal absorption. Pre‐existing healthier lifestyle habits among CeD patients could plausibly play a role, though this would not account for the concurrent reduction in HDL and ApoA1, typically considered protective, alongside higher atherogenic ratios. Reduced cholesterol levels in untreated CeD have been reported previously [34, 35, 36], yet data on cardiovascular disease risk among seropositive untreated patients remain conflicting [37, 38], with a possible elevated risk suggested to arise from chronic inflammation [39, 40, 41, 42]. Importantly, CeD patients on a long‐term gluten‐free diet did not exhibit a worse metabolic profile compared to non‐CeD controls, which is reassuring given existing evidence of potential negative dietary effects of a gluten‐free diet on metabolic health [43, 44, 45].

Screen‐detected CeD patients more frequently reported a history of pre‐eclampsia compared with the non‐CeD population, whereas no differences were observed in other pregnancy outcomes or infertility. Previous data regarding the potential increased risk of adverse reproductive and gestational outcomes, including pre‐eclampsia, in individuals with CeD have been contradictory [46, 47, 48, 49]. Notably, antiphospholipid syndrome—one of the risk factors for pre‐eclampsia—has been linked to autoimmune conditions [50, 51], and, although evidence is limited, also to CeD [52]. The observed increased prevalence of pre‐eclampsia among CeD patients found through population‐based screening is concerning and warrants further investigation. Furthermore, the higher prevalence of type 1 diabetes in CeD patients was an expected finding as there is a shared genetic risk between the two conditions. In fact, co‐screening strategies for the conditions have been proposed as a potentially practical and cost‐efficient approach [53].

CeD patients did not show signs of reduced psychological well‐being at either time point, or of reduced quality of life after a long‐term gluten‐free diet, when compared to the non‐CeD population. Findings on a gluten‐free diet are particularly important as the diet can be restrictive and costly, and maintaining it may impose a burden that could reduce quality of life, especially among the often clinically silent screen‐detected patients. Previous studies on this topic are partly inconsistent, with some reporting improved or unchanged quality of life during dietary treatment, while others have shown a reduction [31, 54, 55, 56, 57, 58]. Heterogeneity between the studies may be driven by methodological factors, as well as variations in the availability, cost and reimbursement of gluten‐free products, the organisation of dietetic support, and the social and cultural impact of the diet. Furthermore, varying follow‐up periods after the diagnosis can potentially influence the inconsistency as in the first years the perceived benefits from dietary treatment may outweigh the diet‐related burden. Here, more than a decade after the diagnosis, beyond the initial ‘honeymoon period’, patients are likely habituated to CeD and treatment, supporting the expectation that long‐term quality of life remains stable. However, more studies assessing quality of life in CeD using CeD‐specific parameters among screen‐detected patients, specifically social functioning and burden of dietary treatment, are needed to clarify in detail the possible harms of screening in this regard.

Long‐term follow‐up showed no increase in mortality among CeD patients found through screening from a population‐representative cohort. Previous studies on this topic have yielded inconsistent findings, likely due to heterogeneity in the included CeD populations, diagnostic definitions, and whether cohorts consisted of untreated or treated patients, or both [37, 59, 60, 61, 62, 63, 64, 65]. Moreover, the timing of the study and the diagnostic efficacy likely influence the results, as older studies and settings with substantial underdiagnosis may overrepresent more severe clinical phenotypes, thereby biasing mortality estimates upward. The present findings are consistent with our previous Finnish study, in which all‐cause mortality was not increased regardless of CeD phenotype, although that from lymphoproliferative diseases and disorders of the central nervous system was elevated [66].

## 5 Strengths and Limitations

Key strengths were the population‐representative cohorts, large sample size and the availability of rich individual‐level data. Furthermore, a substantial proportion of the seropositive individuals identified in this cohort were previously shown to have undergone small‐bowel biopsy confirming mucosal damage, lending histological support to the serological case definition applied here. As a limitation, because the Health 2000 and Health 2011 surveys focused on public health conditions rather than specifically on CeD, information on some important CeD‐related aspects, such as symptoms, a family history of CeD, and adherence strictness to a gluten‐free diet, was lacking. Additionally, the available laboratory parameters differed between time points; in particular, changes in iron status could not be assessed. The lack of biopsy data for all patients and use of only one blood draw for antibody analysis could be considered limitations; however, biopsy was offered to all seropositive individuals, with a high proportion of positive findings, and coeliac disease was subsequently confirmed by physician‐reported diagnosis [28]. None of the individuals in our cohort fulfilled the non‐biopsy criterion for diagnosing CeD (TGA levels > 10 times the upper limit of normal). Rather, the purpose of using double‐seropositivity as our criterion was to identify, with sufficient accuracy, those who truly had the disease. In addition, double‐seropositivity is regarded as highly specific and has been used in previous CeD studies [5, 29, 67, 68]. Furthermore, the diagnostic practice is moving increasingly towards a serology‐based approach [69]. Finally, the questionnaires used to evaluate psychological health and quality of life were not specifically designed to assess social functioning and the burden of dietary treatment in CeD and may not capture disease‐specific impairments in wellbeing. Regarding generalisability, CeD is common and well‐recognised in Finland where maintaining a gluten‐free diet may be easier than in some other countries.

## 6 Conclusions

The present study demonstrated that unrecognised CeD patients exhibited signs of iron deficiency and a higher prevalence of anaemia, while a gluten‐free diet was not associated with deterioration in long‐term quality of life or metabolic health. Notably, even in a high‐prevalence country with active case finding and risk‐group screening, a considerable proportion of individuals with CeD appears to remain unidentified. Our findings demonstrate a favourable benefit‐harm balance for population‐based screening, though further research is needed to determine the treatment‐related burden, to assess how these findings translate to other countries, and to identify optimal, cost‐effective screening strategies [70].

## Author Contributions

**Heini Huhtala:** formal analysis, writing – review and editing. **Iida Ahonen:** formal analysis, writing – original draft, methodology, writing – review and editing. **Katri Kaukinen:** conceptualization, methodology, supervision, writing – review and editing. **Kalle Kurppa:** conceptualization, methodology, supervision, writing – original draft, writing – review and editing. **Laura Kivelä:** conceptualization, funding acquisition, writing – review and editing, writing – original draft. **Juha Taavela:** conceptualization, methodology, data curation, formal analysis, supervision, funding acquisition, writing – original draft, writing – review and editing.

## Funding

This study was financially supported by the Competitive State Research Financing of the Expert Responsibility Areas of Tampere University Hospital, the Finnish Paediatric Foundation, Päivikki and Sakari Sohlberg Foundation, Emil Aaltonen Foundation, Finnish Medical Foundation (7850), The Research Council of Finland (339183, 361421) and the Finnish cultural foundation, the Sigrid Juselius Foundation, the Finnish Celiac Society, and Tampere University Hospital Research Foundation. The funders had no role in the design of the study and data collection, analysis and interpretation of the results, or writing of the manuscript.

## Ethics Statement

The Health 2000 survey was approved by the Ethics Committee for Research in Epidemiology and Public Health in May 2000, while the Health 2011 survey received approval from the Coordinating Ethics Committee in March 2011, which was part of the Hospital District of Helsinki and Uusimaa at that time. For this specific study, a biobank approval was attained from the Biobank maintained by THL (THLBB2024 33).

## Consent

Written informed consent was obtained from all participants. All study data were analysed pseudo‐anonymised without personal information by which the participants could be identified.

## Conflicts of Interest

Outside of the work submitted here, Laura Kivelä has received travel grant from ISSCD and Takeda and Kalle Kurppa personal lecture fees from the Finnish Celiac Society, Columbia University and Thermo Fisher Scientific, and consulting fees from Takeda. Juha Taavela has been a consultant for Falk Pharma, Teva pharmaceuticals, Anaptysbio, Medpace/ForteBio and PathAi and received personal lecture fees from the Finnish Celiac Society. Other study authors declare no conflicts of interest.

## Supporting information


**Table S1:** Demographics, health care utilisation, lifestyle factors, physical measurements, and psychological health in screen‐detected coeliac disease (CeD) patients (*n* = 92) compared to non‐CeD population (*n* = 6216) at baseline in 2000.

**Table S2:** Comparison of background characteristics between baseline cohort (*n* = 6308) and follow‐up cohort (*n* = 3276) at initial screening in 2000.

**Table S3:** Psychosocial health in screen‐detected coeliac disease (CeD) patients following (*n* = 50) and not following (*n* = 8) a gluten‐free diet (GFD) in 2011.

## Data Availability

The data that support the findings of this study are available from Finnish Institute for Health and Welfare. Restrictions apply to the availability of these data, which were used under license for this study. Data are available from the author(s) with the permission of Finnish Institute for Health and Welfare.
