# Supplementary material for: Screen‐Detected Coeliac Disease in a Nationally Representative Cohort: Health Indicators, Long‐Term Outcomes and Mortality
Source: Aliment Pharmacol Ther. 2026 Aug 10;64(9):1248–59. doi: 10.1111/apt.70917 (PMC13651919; doi:10.1111/apt.70917)
Supplement: Supplementary file 1 [file APT-64-1248-s001.docx]

| **Supplementary Table 1.** Demographics, health care utilization, lifestyle factors, physical measurements and psychological health in screen-detected coeliac disease (CeD) patients (n=92) compared to non-CeD population (n=6,216) at baseline in 2000. | | | | | |
| --- | --- | --- | --- | --- | --- |
|  | | **CeD**  **N=92** | **Non-CeD**  **N=6,216** | **P value**^1^ | **P value**^2^ |
| **Demographics** | |  |  |  |  |
| Age, y, median (Q1, Q3) | | 48 (42, 58) | 51 (41, 64) | 0.159 |  |
| Female, % | | 62 | 55 | 0.205 |  |
| Education level, % | |  |  | 0.211 |  |
|  | *Basic^3^* | 39 | 41 |  |  |
|  | *Secondary^4^* | 39 | 31 |  |  |
|  | *Higher^5^* | 22 | 28 |  |  |
|  |  |  |  |  |  |
| **Health care utilization**^6^ | | **%** | **%** |  |  |
| At least one doctor’s visit | | 72 | 74 | 0.549 | 0.480 |
| Use of mental health services | | 3 | 6 | 0.488 | 0.275 |
|  | |  |  |  |  |
| **Lifestyle** | |  |  |  |  |
| Smoking regularly | | 11 | 22 | **0.014** | **0.011** |
| Smoking at least for one year | | 43 | 54 | 0.083 | 0.086 |
| Alcohol consumption, g/week, median (Q1, Q3) | | 12 (0, 55) | 20 (0, 81) | 0.334 | 0.135 |
| Use of prescription medicine^6^ | | 74 | 79 | 0.246 | 0.245 |
| Use of non-prescription medicine^6^ | | 83 | 80 | 0.693 | 0.924 |
|  | |  |  |  |  |
| **Physical measurements** | | **Median (Q1, Q3)** | **Median (Q1, Q3)** |  |  |
| Body mass index, kg/m^2^ | | 26 (24, 29) | 26 (24, 30) | 0.567 | 0.557 |
| Waist circumference, cm | | 90 (82, 98) | 93 (83, 102) | 0.099 | 0.336 |
| Systolic blood pressure, mmHg | | 131 (118, 144) | 132 (120, 148) | 0.225 | 0.526 |
| Diastolic blood pressure, mmHg | | 81 (72, 88) | 82 (74, 90) | 0.234 | 0.204 |
|  | |  |  |  |  |
| **Laboratory parameters** | |  |  |  |  |
| Haemoglobin, g/L | | 140 (129, 150) | 143 (133, 152) | **0.046** | **0.013** |
| Ferritin, ng/mL | | 29 (10, 76) | 68 (32, 128) | **<0.001** | **0.004** |
| GGT, U/L | | 19 (14, 29) | 24 (17, 38) | **<0.001** | **0.036** |
| S-D-25, nmol/L | | 39 (31, 52) | 43 (33, 55) | 0.257 | 0.538 |
| Creatinine, μmol/L | | 68 (60, 74) | 71 (62, 81) | **0.002** | 0.053 |
| TSH, mU/L | | 1.4 (0.9, 2.0) | 1.4 (1.0, 2.0) | 0.619 | 0.511 |
| TC, mmol/L | | 5.4 (4.7, 5.9) | 5.9 (5.2, 6.6) | **<0.001** | **<0.001** |
| HDL-C, mmol/L | | 1.1 (0.9, 1.3) | 1.3 (1.1, 1.6) | **<0.001** | **<0.001** |
| sdLDL-C, mmol/L | | 3.5 (3.0, 4.3) | 3.8 (3.1, 4.5) | **0.024** | 0.060 |
| TC/HDL-C ratio | | 5.1 (3.8, 6.3) | 4.5 (3.6, 5.7) | 0.051 | 0.085 |
| TG, mmol/L | | 1.2 (0.9, 1.7) | 1.3 (1.0, 1.9) | 0.070 | 0.566 |
| ApoA1, g/L | | 1.4 (1.2, 1.5) | 1.6 (1.4, 1.8) | **<0.001** | **<0.001** |
| ApoB, g/L | | 1.2 (0.9, 1.3) | 1.2 (1.0, 1.4) | **0.009** | **0.011** |
| ApoB/ApoA1 ratio | | 0.9 (0.7, 1.0) | 0.8 (0.6, 1.0) | **0.022** | **0.020** |
|  | |  |  |  |  |
| **Psychological health** | |  |  |  |  |
| GHQ-12, total score | | 0 (0, 2) | 0 (0, 2) | 0.095 | 0.089 |
| BDI, total score | | 4 (1, 8) | 5 (2, 10) | 0.108 | 0.166 |
| ^1^Crude analysis; ^2^Adjusted for age and sex; ^3^Not completed vocational school, matriculation examination or degrees from higher vocational institutions, polytechnics and universities; ^4^Completed vocational school and/or passed matriculation examination; ^5^Degrees from higher vocational institutions, polytechnics and universities; ^6^During previous 12 months. ApoA1, serum apolipoprotein A1; ApoB, serum apolipoprotein B; BDI, Beck Depression Inventory, higher score indicates more depressive symptoms; GGT, gamma-glutamyl transferase; GHQ-12, General Health Questionnaire, higher score indicates more psychological distress; HDL-C, serum high-density lipoprotein cholesterol; sdLDL-C, serum small dense low-density lipoprotein cholesterol; S-D-25, serum 25-hydroxyvitamin D; TC, serum total cholesterol; TG, serum triglycerides; TSH, thyroid stimulating hormone. | | | | | |
