# Supplementary material for: Screen‐Detected Coeliac Disease in a Nationally Representative Cohort: Health Indicators, Long‐Term Outcomes and Mortality
Source: Aliment Pharmacol Ther. 2026 Aug 10;64(9):1248–59. doi: 10.1111/apt.70917 (PMC13651919; doi:10.1111/apt.70917)
Supplement: Supplementary file 2 [file APT-64-1248-s002.docx]

| **Supplementary Table 2**. Comparison of background characteristics between baseline cohort (n=6,308) and follow-up cohort (n=3,276) at initial screening in 2000. | | | |
| --- | --- | --- | --- |
|  | | **Baseline cohort**  **N=6,308** | **Follow-up cohort**  **N=3,276** |
| **Demographics** | |  |  |
| Age, y, median (Q1, Q3) | | 51 (41, 64) | 48 (39, 56) |
| Female, % | | 55 | 55 |
| Education level, % | |  |  |
|  | *Basic^1^* | 40 | 29 |
|  | *Secondary^2^* | 32 | 34 |
|  | *Higher^3^* | 28 | 37 |
|  | |  |  |
| **Health care utilization^4^** | | **%** | **%** |
| At least one doctor’s visit | | 78 | 75 |
| Use of mental health services | | 6 | 6 |
|  | |  |  |
| **Lifestyle** | |  |  |
| Smoking regularly | | 21 | 19 |
| Smoking at least for 1 year | | 54 | 45 |
| Alcohol consumption, g/week, median (Q1, Q3) | | 20 (0, 81) | 28 (4, 93) |
| Use of non-prescription medicine^4^ | | 80 | 84 |
| Use of prescription medicine^4^ | | 79 | 77 |
|  | |  |  |
| **Physical measurements** | | **Median (Q1, Q3)** | **Median (Q1, Q3)** |
| Body mass index, kg/m^2^ | | 26 (24, 30) | 26 (23, 29) |
| Waist circumference, cm | | 93 (83, 102) | 91 (82, 100) |
| ^1^Not completed vocational school, matriculation examination or degrees from higher vocational institutions, polytechnics and universities; ^2^Completed vocational school and/or passed matriculation examination; ^3^Degrees from higher vocational institutions, polytechnics and universities; ^4^During previous 12 months. | | | |
