# Supplementary material for: Screen‐Detected Coeliac Disease in a Nationally Representative Cohort: Health Indicators, Long‐Term Outcomes and Mortality
Source: Aliment Pharmacol Ther. 2026 Aug 10;64(9):1248–59. doi: 10.1111/apt.70917 (PMC13651919; doi:10.1111/apt.70917)
Supplement: Supplementary file 3 [file APT-64-1248-s003.docx]

| **Supplementary Table 3.** Psychosocial health in screen-detected coeliac disease (CeD) patients following (n=50) and not following (n=8) a gluten-free diet (GFD) in 2011. | | | |
| --- | --- | --- | --- |
|  | **GFD**  **N=50** | **Non-GFD**  **N=8** | **P value** |
| EUROHIS-QoL, median (Q1, Q3) | 4.1 (3.8, 4.4) | 4.3 (4.1, 4.5) | 0.281 |
| GHQ-12, median (Q1, Q3) | 0 (0, 1) | 0 (0, 1) | 0.603 |
| Self-reported current health status as “good” or “fairly good”, % | 82 | 100 | 0.334 |
| EUROHIS-QOL, higher score indicates better quality of life; GHQ-12, General Health Questionnaire, higher score indicates more psychological distress. | | | |
